# Axially Coordinated Gold Nanoclusters Tailoring Fe–N–C Nanozymes for Enhanced Oxidase‐Like Specificity and Activity

**DOI:** 10.1002/advs.202306911

**Published:** 2024-01-09

**Authors:** Yameng Xie, Fuli Sun, Kuan Chang, Guang Li, Zhijia Song, Jiayu Huang, Xiqing Cheng, Guilin Zhuang, Qin Kuang

**Affiliations:** ^1^ State Key Laboratory of Physical Chemistry of Solid Surfaces, Collaborative Innovation Center of Chemistry for Energy Materials, Department of Chemistry, College of Chemistry and Chemical Engineering Xiamen University Xiamen 361005 China; ^2^ College of Chemical Engineering Zhejiang University of Technology Hangzhou 310032 China; ^3^ School of Chemical and Environmental Engineering Shanghai Institute of Technology Shanghai 201418 China

**Keywords:** AChE detection, Fe‐N‐C SACs, Gold nanoclusters, nanozymes, Oxidase‐like activity

## Abstract

Metal–organic frameworks (MOF) derived nitrogen‐doped carbon‐supported monodisperse Fe (Fe–N–C) catalysts are intensively studied, but great challenges remain in understanding the relationship between the coordination structure and the performance of Fe–N–C nanozymes. Herein, a novel nanocluster ligand‐bridging strategy is proposed for constructing Fe‐S_1_N_4_ structures with axially coordinated S and Au nanoclusters on ZIF‐8 derived Fe–N–C (labeled Au_x_/Fe‐S_1_N_4_‐C). The axial Au nanoclusters facilitate electron transfer to Fe active sites, utilizing the bridging ligand S as a medium, thereby enhancing the oxygen adsorption capacity of composite nanozymes. Compared to Fe‐N‐C, Au_x_/Fe‐S_1_N_4_‐C exhibits high oxidase‐like specificity and activity, and holds great potential for detecting acetylcholinesterase activity with a detection limit of 5.1 µU mL^−1^, surpassing most reported nanozymes.

## Graphical abstract

A novel nanocluster ligand‐bridging strategy is proposed to construct Fe–S_1_N_4_ structure with axially coordinated S and Au nanoclusters on ZIF‐8 derived Fe–N–C. This well‐designed Au_x_/Fe‐S_1_N_4_‐C nanozyme exhibit high oxidase‐like activity and specificity due to its better oxygen adsorption capacity.

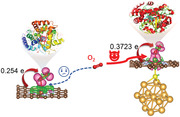

## 1 Introduction

Nanozymes, a class of simple and efficient alternative nanomaterials with enzyme‐like catalytic activity, have attracted increasing attention because of their inherent advantages of high stability, low cost, and remarkable potential for diverse applications such as biosensors, immunoassays, and disease therapies.^[^ ^1^ ^]^ Among all nanozymes, metal−organic frameworks (MOF) derived nitrogen‐doped carbon supported monodisperse Fe (Fe–N–C) catalysts have been intensively studied because Fe‐N_x_ active sites closely resemble natural enzymes.^[^ ^2^ ^]^ Many researchers are committed to studying the intricate relationship between the coordination structure and performance of Fe–N–C nanozymes. For example, Fe‐N_4_ single‐atom catalysts (SACs)^[^ ^2b^ ^]^ and Fe‐N_3_P SACs^[^ ^2c^ ^]^ with peroxidase‐like catalytic activity and Fe‐N_5_ SA/CNFs^[^ ^3^ ^]^ with oxidase‐like catalytic activity have been synthesized by controlling the geometric and electronic structures of Fe active centers. Although great progress has been achieved, challenges persist in understanding the interplay between Fe‐N_x_ coordination structures and catalytic selectivity.

In natural enzymes, horseradish peroxidase structurally has an imidazole axial ligand environment, which plays a crucial role in facilitating peroxide O─O bond cleavage.^[^ ^4^ ^]^ Additionally, Cytochrome P‐450 presents an axial Fe─S bond, enabling electron donation from the axial thiolate ligand for O_2_ substrates.^[^ ^5^ ^]^ These distinctive features underscore the essential role of axial electron donation from non‐metallic S atoms in determining the specificity and activity. Drawing inspiration from these prototypical enzymes, we propose a novel strategy of incorporating an axial S coordination structure onto Fe‐N_4_ sites commonly associated with peroxidase activity, which holds great promise for the selectivity regulation of Fe‐N‐C based nanozymes.

Our approach centers around a nanocluster ligand‐bridging strategy aimed at constructing Fe‐S_1_N_4_ structures with axially coordinated S and Au nanoclusters to mimic the axial coordination structure of the natural P450. In this strategy, we leveraged the thiolate (SR) ligands, L‐Cysteine (L‐Cys), in Au nanoclusters (Au_25_(SR)_18_) due to its strong bonding ability with Fe, serving as a bridging agent to connect Au nanoclusters to the Fe‐N_4_ active sites. This connection facilitates the transfer of more electrons to active sites.^[^ ^6^, ^7^ ^]^ To our great excitement, compared to Fe–N–C, the as‐synthesized Au_x_/Fe‐S_1_N_4_‐C exhibited remarkable oxidase‐like specificity and activity, along with promising potential in acetylcholinesterase (AChE) detection.

## 2 Results and Discussion

### 2.1 Structure Characterization

**Figure 1:**
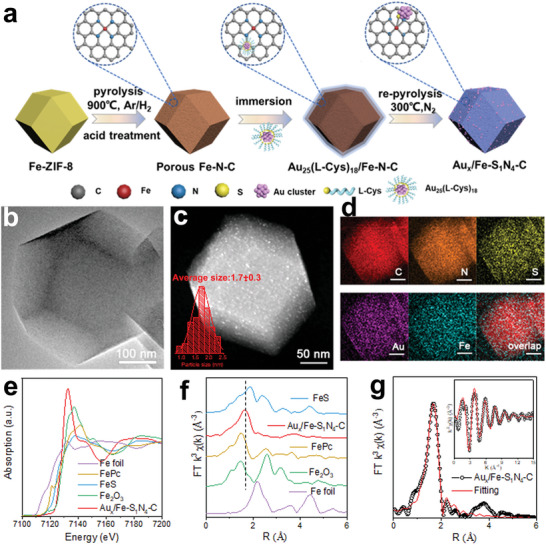
Synthesis and characterizations of Au_x_/Fe‐S_1_N_4_‐C. a) Schematic preparation process. b) TEM and c) HAADF‐STEM images. Inset in (c) is corresponding particle‐size histograms of Au nanoclusters supported on Fe‐N‐C. d) EDS elemental mapping. e) Normalized XANES spectra at Fe K‐edge and f) the corresponding k^3^‐weighted Fourier‐transformed spectra. g) Fitting curves of the EXAFS in the r‐space and k‐space (inset of (g)).

As illustrated in **Figure** **1**a, to construct Au_x_/Fe‐S_1_N_4_‐C nanozymes, negatively charged Au_25_(L‐Cys)_18_ (denoted as Au_25_) nanoclusters were anchored onto positively charged ZIF‐8 derived Fe‐N‐C matrix of rhombohedral dodecahedron morphology through impregnation and pyrolysis at given temperature (Figures S1and S2, Supporting Information). For convenience, the final samples were labeled as Au_x_/Fe‐N‐C‐T (where T represents the pyrolysis temperature). The sample obtained at 300 °C was specially designated as Au_x_/Fe‐S_1_N_4_‐C to highlight the coordination structure of its active sites.

Figure 1b shows a representative bright‐field transmission electron microscopy (TEM) image of Au_x_/Fe‐S_1_N_4_‐C. In contrast to Au_25_/Fe‐N‐C without undergoing re‐pyrolysis (Figure S3, Supporting Information), highly dispersed and uniformly sized Au nanoclusters were more clearly observed on the Fe‐N‐C matrix, as revealed in the high‐angle annular dark‐field scanning transmission electron microscopy (HAADF‐STEM) image of Au_x_/Fe‐S_1_N_4_‐C (Figure 1c). Remarkably, the average size of Au nanoclusters was only 1.7 nm, akin to the pristine Au_25_(L‐Cys)_18_. Furthermore, aberration‐corrected high‐angle annular dark‐field scanning transmission electron microscopy (AC‐HAADF‐STEM, Figure S4, Supporting Information) shows that pyrolysis temperature of 300°C has little effect on the Au core of the clusters and does not produce individual atoms of Au. Elemental mapping confirmed the uniform distribution of Fe, Au and S element on the Fe‐N‐C matrix, corroborating the high dispersion of Au nanoclusters (Figure 1d). Thermal gravity and differential thermal analysis (Figure S5, Supporting Information) revealed that Au_25_ nanoclusters experienced partial decomposition during pyrolysis at 300 °C, which resulted in some of the S atoms in the L‐Cys ligands bonding to Fe–N–C matrix, thereby enhancing the interaction between Au_25_ nanoclusters and Fe–N–C matrix. Moreover, the graphitization degree of Fe–N–C matrix was improved to some extent (Figure S6, Supporting Information).

X‐ray absorption fine structure (XAFS) analysis was used to further probe into the chemical state and coordination structure of Fe sites within Au_x_/Fe‐S_1_N_4_‐C. The analysis on Fe K‐edge x‐ray absorption near‐edge structure (XANES) of Au_x_/Fe‐S_1_N_4_‐C

(Figure 1e) suggested that the average oxidation state of Fe in Au_x_/Fe‐S_1_N_4_‐C was between +2 and +3. The Fourier‐transformed k^3^‐weighted extended XAFS (FT‐EXAFS) spectrum of Au_x_/Fe‐S_1_N_4_‐C at Fe K‐edge presented a main peak at 1.63 Å, FePc at 1.50 Å and Fe‐S bond at 1.87 Å, while no Fe─O bond (1.47 Å) or Fe─Fe bond (2.18 Å) was detected (Figure 1f; Table S1, Supporting Information). Besides, the wavelet transforms (WT) contour plot (Figure S7, Supporting Information) of Au_x_/Fe‐S_1_N‐C exhibited higher intensity maximum (5.2 Å) than FePc (2.7 Å), which is attributed to the Fe–S bonding. The above results indicate that Fe atoms are atomically dispersed and coordinated by N and S atoms. The corresponding EXAFS fitting of the first coordination shell was performed to ascertain the structural parameters and quantitative chemical configuration of Fe atoms (Figure 1g). The coordination number of Fe atom was close to five, comprising four N atoms and one S atom (Table S1, Supporting Information), further affirming the formation of Fe‐S_1_N_4_ moieties in Au_x_/Fe‐S_1_N_4_‐C. The above analysis indicated that Au_x_/Fe‐S_1_N‐C was characterized by Fe‐S_1_N_4_ sites, where Au_x_ nanoclusters were anchored onto Fe‐N_4_ sites through the axial coordination of S atoms with Fe‐N_4_.

**Figure 2:**
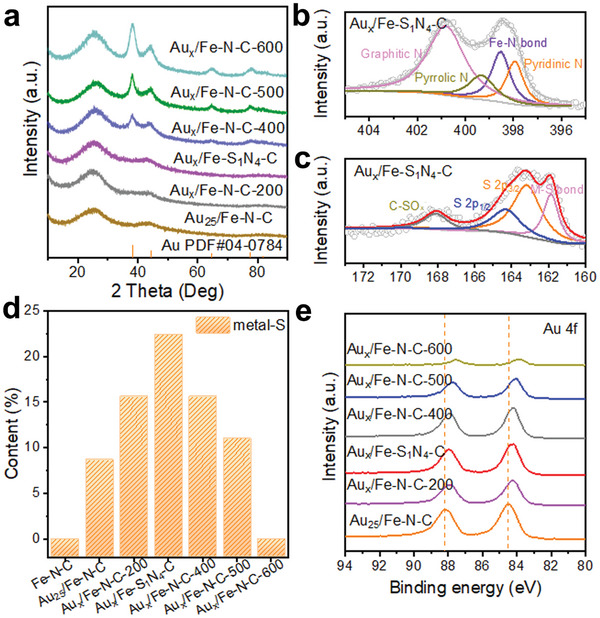
Characterizations of Au_25_/Fe‐N‐C and Au_x_/Fe‐N‐C‐X obtained with different re‐pyrolysis temperatures. a) XRD patterns. b) High‐resolution XPS spectra of N 1s and c) S 2p of Au_x_/Fe‐S_1_N_4_‐C. d) Metal‐S contents calculated by XPS spectra. (e) XPS spectra of Au 4f.

Of note, the re‐pyrolysis temperature exerted a significant impact on the size and distribution of the Au_x_ species anchored onto the Fe–N–C matrix. With the re‐pyrolysis temperature increasing progressively from 300 °C to 400, 500, and 600 °C, the average particle size of Au species increased from 1.7 nm to 3.4, 3.9, and 5.1 nm, correspondingly (Figure S8, Supporting Information). Once the re‐pyrolysis temperature exceeded 400 °C, characteristic diffraction peaks attributed to metallic Au became discernible in the X‐ray diffraction (XRD) patterns of Au_x_/Fe–N–C–T samples, and their intensity increased with the rise of temperature (**Figure** **2**a). To reveal the influence of the re‐pyrolysis on the chemical structure and electron structure of Fe–N–C matrix as well as Au_x_ species anchored on Fe–N–C matrix, X‐ray photoelectron spectroscopy (XPS) analysis were conducted. The metal content of each sample was similar (Table S2, Supporting Information). The N 1s XPS spectrum of Au_x_/Fe‐S_1_N_4_‐C can be deconvoluted to pyridinic‐N (397.9 eV), Fe‐N (398.5 eV), pyrrolic‐N (399.3 eV), and graphitic‐N (400.8 eV) species (Figure 2b). Furthermore, the interconversion and stability of the structure with increasing temperature ^[^ ^8^ ^]^ resulted in an increase in the graphitic nitrogen content, a decrease in the pyrrolic nitrogen content, and an initial increase and then a decrease in the pyridinic nitrogen (Figure S9 and Table S3, Supporting Information). The metal‐S (Fe–S and Au–S) species at 161.9 eV appeared in the S 2p XPS spectrum of Au_x_/Fe‐S_1_N_4_‐C (Figure 2c). Interestingly, the proportion of metal‐S bonds increased and then decreased with the increase of the re‐pyrolysis temperature, and Au_x_/Fe‐S_1_N_4_‐C presented the highest ratio of metal‐S bonds (Figure 2d, see Figure S10 and Table S4, Supporting Information for details). On the other hand, the Au 4f peak gradually shifted to lower binding energies with increasing re‐pyrolysis temperature (Figure 2e). This indicated that the loss of thiolate ligands leads to the reduction of positive monovalent Au on the surface to zero valence, and the Au─S bond weakened with re‐pyrolysis temperature elevation. Taking all the results together, the increase of metal‐S bond in the Au_x_/Fe‐S_1_N_4_‐C should be attributed to the increase of Fe─S bond. That's to say, S achieves optimal binding to Au and Fe (Au─S─Fe bonds) at 300 °C.

### 2.2 Specific Oxidase‐Like Activity Tests

**Figure 3:**
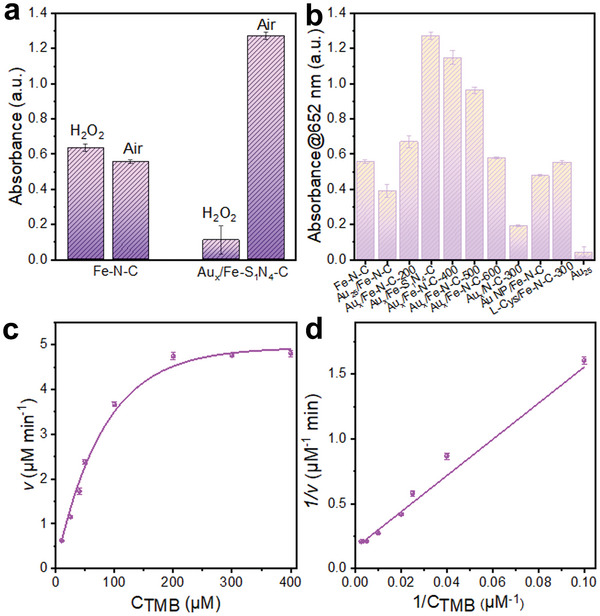
Mimic enzyme performance of Fe‐N‐C and Au_x_/Fe‐N‐C‐T obtained with different re‐pyrolysis temperatures. a) Comparison of peroxidase‐like and oxidase‐like activities. b) The absorbance of different samples at 652 nm. c) Steady‐state kinetic assay toward TMB. d) Michaelis‐Menten curves toward TMB.

The peroxidase and oxidase‐like activities of both Fe–N–C and Au_x_/Fe‐S_1_N_4_‐C were tested with 3,3′,5,5′‐tetramethylbenzidine (TMB) as the chromogenic substrate (**Figure** **3**a; Figures S11 and S12, Supporting Information). While the peroxidase activity of Fe‐N‐C marginally exceeded its oxidase activity, Au_x_/Fe‐S_1_N_4_‐C exhibited the remarkable selectivity for oxidase‐like activity. The oxidase‐like activity Au_x_/Fe‐S_1_N_4_‐C was 12 times that of its peroxidase activity. To reveal the role of Fe‐N_4_ sites and Au nanoclusters, the oxidase‐like activities of the Fe‐free Au_x_/N‐C‐300 (Figure S13, Supporting Information), Au NP/Fe‐N‐C (Figure S14, Supporting Information), L‐Cys/Fe‐N‐C‐300 (Figure S15, Supporting Information), Au_25_ and the Au_x_/Fe‐N‐C‐T samples obtained at different re‐pyrolysis temperatures (i.e., 200, 300, 400, 500, and 600 °C) were also tested (Figure 3b; Figure S16, Supporting Information). Among all the samples tested, Au_x_/Fe‐S_1_N_4_‐C obtained at 300 °C showed the best selectivity and catalytic activity, with a 2.4‐fold and 6.7‐fold increase in oxidase‐like activity compared to Au_x_‐free sample (Fe–N–C) and the Fe‐free sample (Au_x_/N‐C‐300), respectively. The poor activity of Au_x_/N‐C‐300 proved that the active centers are attributed to Fe sites rather than Au_x_. As shown in Figure 3b, Au NP/Fe‐N‐C (Figure S14, Supporting Information) exhibits lower activity compared to Fe‐N‐C due to the partial occupancy of the active site by Au. After calcination at 300 °C, L‐Cys in L‐Cys/Fe‐N‐C‐300 is essentially residue‐free (Figure S15, Supporting Information), resulting in similar oxidase‐like activity to Fe‐N‐C. Notably, Au_25_ shows almost no oxidase‐like activity. Comparative analysis of the oxidase‐like activities of these samples reveals that Au_25_, L‐Cys, and Au NP are not active centers. In addition, the oxidase‐like activities of Au_x_/Fe‐N‐C‐T showed a volcanic relationship with the re‐pyrolysis temperature. Clearly, the re‐pyrolysis temperature exerted a significant effect on the formation of Fe‐S_1_N_4_ axial coordination structure. At 200 °C, insufficient temperature results in too few Au‐S‐Fe bonds being formed. Conversely, excessively high temperature causes agglomeration of Au clusters, as well as breakage of Fe─S and Au─S bonds, resulting in the decline of Au‐S‐Fe bonds. Au_x_/Fe‐S_1_N_4_‐C has the highest amount of Au─S─Fe bonds, similar to the axial Fe─S bonds of cytochrome P‐450, which aids in electron transfer to the Fe active sites. Consequently, the Au_x_/Fe‐S_1_N_4_‐C has the highest activity and selectivity. The Michaelis–Menten constant (*K*_m_) of Au_x_/Fe‐S_1_N_4_‐C was calculated as 0.0879 mm based on the kinetic assay (Figure 3c,d), indicating the good affinity of Au_x_/Fe‐S_1_N_4_‐C toward TMB. In addition, the Au_x_/Fe‐S_1_N_4_‐C exhibited an oxidase‐like catalytic rate of 0.1051 µm s^−1^, surpassing the performance of previously reported Au or Fe based oxidase mimics (**Table** **1**).

**Table 1:** Comparison of the kinetic parameters of the oxidase‐like nanozymes.

Nanozyme	Substrate	K_m_ (mM)	V_max_ [µM s^−1^]	W [µg mL^−1^]	Reference
Au_x_/Fe‐S_1_N_4_‐C	TMB	0.0879	0.1051	1.5	This work
Fe‐SANs	TMB	0.114	0.0651	10	[9]
Cu_3_/ND@G	TMB	2.89	0.115	100	[10]
CuN_5_ SA/CNF	TMB	0.124	4.7×10^−4^	5	[3]
Commercial Pt/C	TMB	0.129	0.052	5	[3]
FeN_5_ SA/CNF	TMB	0.148	0.758	5	[3]
FeN_4_ SA/CNF	TMB	0.143	0.045	5	[3]
Au@HCNs	TMB	0.170	0.049	50	[11]
Pd_12_ Cage	TMB	/	0.0708	/	[12]
Fe‐N‐C SAzymes	TMB	1.81	6.01×10^−4^	17	[13]
N‐PCNSs‐5	TMB	0.095	2.7×10^−3^	25	[14]
N‐PCNSs‐3	TMB	0.084	4.2×10^−3^	25	[14]
QAU‐Z1	TMB	0.102	1.02 ×10^−2^	20	[15]
GNC‐900	TMB	0.22	9.6×10^−3^	20	[15]
Fe‐N‐C‐850	TMB	0.23	0.133	/	[16]
Co‐SAs@NC	TMB	3.48	0.459	160	[17]
Zn_80_Co_20_‐1000	TMB	0.195	0.046	20	[18]
PtCo@G@CPB	TMB	0.0505	0.129	21.265	[19]
MnSA–N_3_–C	TMB	1.17	0.487	0.5	[20]
Fe NP‐NC	TMB	0.095	0.0437	200	[21]
Mn/PSAE	TMB	0.11	0.07	30	[22]

### 2.3 Catalytic Mechanism

**Figure 4:**
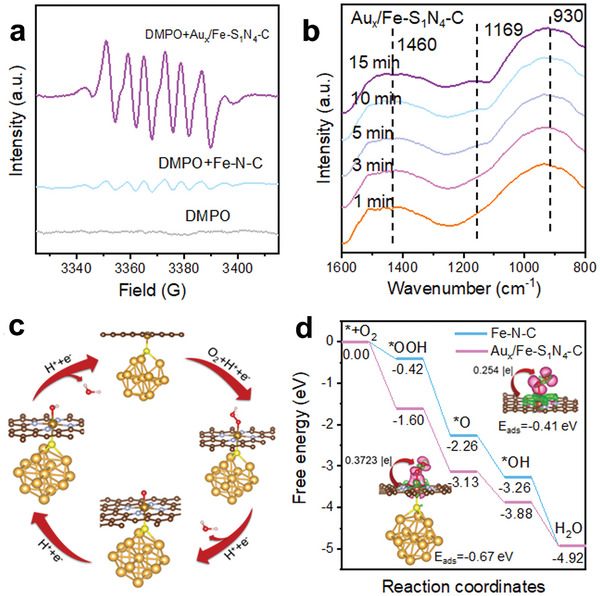
Catalytic Mechanism of Fe‐N‐C and Au_x_/Fe‐S_1_N_4_‐C. a) EPR spectra b) In situ FTIR recorded under an O_2_ atmosphere. c) The reaction pathways of O_2_ reduction to H_2_O. d) Free energy diagram for oxygen reduction. Inset in (d) is structure of O_2_ adsorption configuration, differential charge density and Bader charge. (The purple and green areas show the accumulation and depletion of charges with the iso‐surface value of 1.0 × 10^−4^ e/bohr^3^.The bronze, light blue, brown, yellow, gold, red, and white spheres represent the C, N, Fe, S, Au, O, and H atoms, respectively.).

An in‐depth exploration of the catalytic mechanisms and reaction pathways offers promising insights for the rational design of MOF‐derived Fe‐N‐C nanozymes. The investigation of intermediate radicals was conducted through in situ electron paramagnetic resonance (EPR), using DMPO as a spin trapping agent (**Figure** **4**a). The Au_x_/Fe‐S_1_N_4_‐C exhibited robust characteristic signals corresponding to DMPO‐•O_2_^−^, while the signals from the Fe‐N‐C was much weaker. This observation suggests that Au_x_/Fe‐S_1_N_4_‐C exhibits enhanced capacity in activating O_2_ to generate •O_2_^−^ compared to the Fe–N–C samples. To further elucidate the O_2_ activation at the Au_x_/Fe‐S_1_N_4_ sites, in situ fourier transform infrared (FTIR) spectroscopy was employed. In the presence of O_2_, the signal at 1100 cm^−1^ on Fe‐N‐C gradually increased, attributed to ^*^OH (Figure S17, Supporting Information). For the Au_x_/Fe‐S_1_N_4_‐C (Figure 4b), the absorption peak exhibited a subtle shift compared with Fe–N–C, indicating that the formation of Au_x_/Fe‐S_1_N_4_ sites affects the electronic structure of Fe active sites. According to the literature, the absorption peaks at 930, 1169, and 1460 cm^−1^ represented the adsorption of ^*^O_2_, ^*^OOH, ^*^O_2_^−^ on Fe‐S_1_N_4_ sites,^[^ ^23^ ^]^ respectively, which was consistent with the EPR results. This affirms that the catalytic mechanism of Au_x_/Fe‐S_1_N_4_‐C diverges from that of Fe–N–C. The introduction of Au_x_ cluster‐S by axial coordination with Fe–N_4_ changes the electronic structures of Fe–N–C catalyst, which endows Au_x_/Fe–S_1_N_4_–C with superior oxygen adsorption capacity compared to Fe‐N‐C, thus exhibiting excellent oxidase activity and selectivity.

To understand the role of Au_x_‐S axial coordination in enhancing the activity of Au_x_/Fe–S_1_N_4_–C oxidase‐like enzymes, we investigated the adsorption energies and associated reaction barrier via density‐functional theory calculations. Given that the metal core of the Au_25_ cluster is Au_13_,^[^ ^24^ ^]^ which has the greatest impact on the electronic structure of S, and considering the computational requirements, we employed Au_13_ as the representative model to present Au_x_ species formed on the Fe–N–C after re‐pyrolysis (Figure 4c; Figure S18, Supporting Information). Significantly, the rate‐determining step for Fe‐N‐C corresponded to the initial process (O_2_ + H^+^+ e^−^ →^*^OOH) due to the weak O_2_ adsorption on Fe‐N‐C (E_ads_ = −0.41 eV, charge = 0.254 |e|) (Figure 4d). In contrast, Au_x_/Fe‐S_1_N_4_‐C exhibited significantly enhanced O_2_ adsorption (E_ads_ = −0.67 eV, charge = 0.3723 |e|) through axial Au_x_‐S coordination Fe sites, promoting four‐electron oxygen reduction. In short, the additional axially‐coordinated Au_x_‐S give Au_x_/Fe‐S_1_N_4_‐C a strong electron push effect, which activates O_2_ and facilitates the cleavage of the O–O band, ultimately leading to significantly enhanced oxidase‐like activity.

### 2.4 Acetylcholinesterase Activity Detection

**Figure 5:**
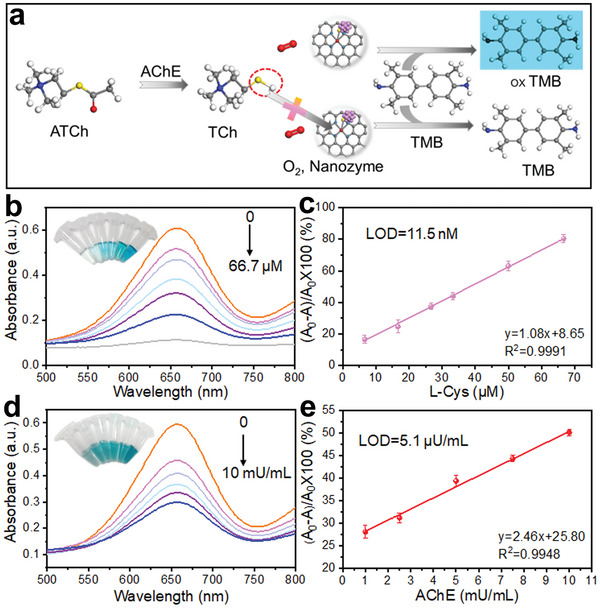
Au_x_/Fe‐S_1_N_4_‐C +TMB system for colorimetric detection. a) Schematic illustration. b) UV–vis spectra in the presence of L‐Cys. c) Linear range for detection of L‐Cys. d) UV–vis spectra in the presence of AChE. e) The linear relationship for detection of AChE.

As a proof‐of‐concept application, the as‐designed highly specific oxidase‐like Au_x_/Fe‐S_1_N_4_‐C was applied to a colorimetric assay for sensitive, rapid and efficient detection of AChE. As shown in **Figure** **5**a, AChE first catalyze the hydrolysis of acetylthiocholine (ATCh) into thiocholine (TCh). TCh as a sulfhydryl molecule tends to coordinate with metal atoms, severely blocking the active sites of Au_x_/Fe‐S_1_N_4_‐C and consequently inhibiting its oxidase‐like activity. It is well‐established that certain sulfhydryl biomolecules, such as L‐Cys and TCh, are directly implicated in several diseases.^[^ ^12^ ^]^ Here, we initially explored the influence of L‐Cys on the activity of Au_x_/Fe‐S_1_N_4_‐C nanozymes. As expected, the absorbance at 652 nm substantially decreased upon L‐Cys addition (Figure 5b). This implies that the active sites of the nanozymes are blocked, and the antioxidant properties of L‐Cys scavenge the free radicals, leading to a reduction in color intensity. The calculated limit of detection (LOD) is as low as 11.5 nM (Figure 5c) according to the 3σ rules (LOD = 3σ/S, where σ is the relative standard deviation (RSD), S is the slope of the calibration curve for the linear range portion), surpassing most reported nanozymes.^[^ ^13^ ^]^

We further investigated the sensitivity of Au_x_/Fe‐S_1_N_4_‐C for the detection of AChE activity in the presence of ATCh (5 mm) across AChE concentrations ranging from 1 to 10 mU mL^−1^. As AChE increased, the activity of Au_x_/Fe‐S_1_N_4_‐C decreased, resulting in lower absorbance values (Figure 5d). The relationship between calculated absorbance variations and the AChE activity is described in Figure 5e. The results indicate that the Au_x_/Fe‐S_1_N_4_‐C based biosensor can be used to assess AChE activity with a LOD of 5.1 µU mL^−1^. Clearly, the Au_x_/Fe‐S_1_N_4_‐C+TMB system exhibited superior performance compared to previous reports (Table S5, Supporting Information).

Furthermore, a series of potential interfering substances were used to examine the anti‐interference ability of the biosensor (Figure S20, Supporting Information). Negligible changes were observed in the detection of AChE. These results highlight the promising application of Au_x_/Fe‐S_1_N_4_‐C in AChE activity detection. Sustaining stability and obtaining reproducible experimental results are crucial factors for achieving practical applications of the AChE biosensor. As depicted in Figure S21 (Supporting Information), the oxidase‐like activity of the AChE biosensor slightly decreased after storage at room temperature, indicating its remarkable stability. Furthermore, the RSD of five independently prepared AChE biosensors was 3.06%. These results demonstrate that the developed Au_x_/Fe‐S_1_N_4_‐C oxidase‐like nanozymes‐based sensor has excellent long‐term stability and reproducibility, making it highly potential for practical applications.

To confirm the feasibility of the proposed method in real biological samples, we applied the standard addition method to detect L‐Cys (Table S6, Supporting Information) and AChE (Table S7, Supporting Information) activity using diluted normal human serum (5%) as the matrix.^[^ ^25^ ^]^ The different concentrations of L‐Cys and AChE solutions were added to the two diluted serum samples, respectively. It can be noticed that the method detection of L‐Cys and AChE activity exhibited satisfactory recovery ranging (100.21‐99.66%, 101.00‐99.67%) and low RSD (0.13‐0.60%, 0.25‐1.98%). This suggests that our colorimetric assay has significant potential for application in real samples.

## 3 Conclusion 

In summary, a novel Au_x_/Fe–S_1_N_4_‐C nanozyme containing Fe–S_1_N_4_ structures with axially coordinated S and Au cluster was successfully constructed via our proposed cluster ligand‐bridging strategy. Our study demonstrates that the axial Au nanoclusters facilitate electron transfer to Fe sites via the bridging ligand S, greatly enhancing the oxygen adsorption capacity of Au_x_/Fe–S_1_N_4_–C. Consequently, compared to Fe–N–C, Au_x_/Fe–S_1_N_4_–C exhibited remarkable selectivity, with an oxidase‐like activity 12 times higher than its peroxidase. Notably, Au_x_/Fe–S_1_N_4_–C demonstrated a lower LOD and better anti‐interference ability in AChE detection, which has great potential for application in real samples. This work helps us to understand the structure‐selectivity relationship of peroxidase and oxidase‐like enzymes, laying the foundation for the rational design and realization of highly selective oxidase‐like nanozymes.

## 4 Experimental Section

### Synthesis of Fe–N–C

Typically, zinc nitrate hexahydrate (2.38 g, 8 mmol) and ferrocene (0.465 g, 2.5 mmol) were dissolved in methanol (60 mL). The solution was then mixed with a solution of 2‐methylimidazole (2.628 g, 15 mmol) in methanol (60 mL). The resulting mixture was stirred for 6 h at room temperature. Afterward, Fe@ZIF‐8 was obtained by centrifugation and washed thoroughly with methanol, and dried at 70 °C under vacuum. The Fe@ZIF‐8 power was transferred into a ceramic boat, placed in a tube furnace, and heated to 900 °C for 2 h with a heating rate of 5 °C min^−1^ under H_2_/Ar (5%:95%), followed by natural cooling to room temperature. The resulting powder was then washed in 0.5 mol L^−1^ sulfuric acid at 80°C for 10 h to remove unstable species.

### Synthesis of Au_x_/Fe–N–C–T

To form L‐Cys‐Au(I) complexes, aqueous solutions of HAuCl_4_ (23.4 mm, 0.4 mL) and L‐Cys (5 mm, 4 mL) were mixed in water (4.7 mL). An aqueous NaOH solution (1 M, 0.1 mL) was then introduced into the reaction mixture, followed by the addition of 0.2 mL of NaBH_4_ solution (prepared by dissolving 43 mg of NaBH_4_ powder in 10 mL of 0.2 m NaOH solution). The L‐Cys ‐Au_25_ nanoclusters were obtained after 3 h for further synthesis. Subsequently, 25 mg Fe–N–C were dissolved in water (5 mL), which was added to the above L‐Cys‐Au_25_ aqueous solution. After stirring for 20 h at room temperature, the Au_25_/Fe–N–C was obtained by centrifugation, washed thoroughly with water, and dried at 60 °C under vacuum. The resulting Au_25_/Fe–N–C power was then transferred into a ceramic boat and heated to 200–600 °C for 2 h with a heating rate of 5 °C min^−1^ under N_2_, followed by natural cooling to room temperature.

## Conflict of interest

The authors declare no conflict of interest.

## Supporting information

Supporting Information

## Data Availability

The data that support the findings of this study are available from the corresponding author upon reasonable request.
