# Supplementary material for: Axially Coordinated Gold Nanoclusters Tailoring Fe–N–C Nanozymes for Enhanced Oxidase‐Like Specificity and Activity
Source: Adv Sci (Weinh). 2024 Jan 9;11(11):2306911. doi: 10.1002/advs.202306911 (PMC10953587; doi:10.1002/advs.202306911)
Supplement: Supplementary file 1 — Supporting Information [file ADVS-11-2306911-s001.pdf]

ADVANCED SCIENCE
Open Access

Supporting Information

for *Adv. Sci.*, DOI 10.1002/advs.202306911

Axially Coordinated Gold Nanoclusters Tailoring Fe–N–C Nanozymes for Enhanced Oxidase-Like Specificity and Activity

Yameng Xie, Fuli Sun, Kuan Chang, Guang Li, Zhijia Song, Jiayu Huang, Xiqing Cheng, Guilin Zhuang* and Qin Kuang**

Supporting Information

Axially coordinated Gold Nanoclusters Tailoring Fe-N-C nanozymes for Enhanced Oxidase-like Specificity and Activity

Yameng Xie, Fuli Sun, Kuan Chang, Guang Li, Zhijia Song, Jiayu Huang, Xiqing Cheng,* Guilin Zhuang,* Qin Kuang*

---

[*] Y. M. Xie, K. Chang, G. Li, Z.J. Song, J.Y. Huang, Prof. Q. Kuang

State Key Laboratory of Physical Chemistry of Solid Surfaces, Collaborative Innovation Center of Chemistry for Energy Materials, Department of Chemistry, College of Chemistry and Chemical Engineering

Xiamen University

Xiamen 361005 (China).

E-mail: qkuang@xmu.edu.cn

F. L. Sun, Prof. G. L. Zhuang

College of Chemical Engineering

Zhejiang University of Technology

Hangzhou 310032 (China)

E-mail: glzhuang@zjut.edu.cn

Dr. X.Q. Cheng

School of Chemical and Environmental Engineering

Shanghai Institute of Technology

Shanghai 201418(China)

E-mail: xiqingcheng@sit.edu.cn

1. Material and methods

1.1. Chemicals and materials

All chemical reagents were used as received without further purification. Hydrogen tetrachloroaurate (III) hydrate ($HAuCl_4·3H_2O$), 3,3′,5,5′ tetramethylbenzidine (TMB) and L-glutathione (reduced, GSH) were obtained from Alfa Aesar. Acetylcholinesterase (AChE), sodium borohydride ($NaBH_4$) and L-cysteine (L-Cys) were purchased from Sigma-Aldrich; sodium hydroxide (NaOH) was gained from Macklin Biochemical Co., Ltd. (China). Zinc nitrate hexahydrate (Zn $(NO_3)_2·6H_2O$), sulfuric acid (H_2SO_4), methanol (CH_3OH), ferrocene, and 2-methylimidazole were supplied by J.K. Magnesium chloride ($MgCl_2$), calcium chloride ($CaCl_2$), nickel (II) nitrate hexahydrate (Ni $(NO_3)_2·6H_2O$), acetic acid ($C_2H_4O_2$), sodium acetate (NaAc), hydrogen peroxide (H_2O_2, 30%), sodium chloride (NaCl), potassium chloride (KCl), calcium acetate monohydrate ($Ca(Ac)_2·H_2O$), D-(+)-glucose anhydrous ($C_6H_{12}O_6$, glucose), sucrose ($C_{12}H_{22}O_{11}$), L-proline ($C_5H_9NO_2$, Pro) and dimethyl sulfoxide (C_2H_6OS, DMSO) were purchased from Sinopharm Chemical Reagent Co. Ltd (Shanghai, China). Glucose oxidase (GOx), tyrosinase (TYR), lysozyme, and trypsin were purchased from Sigma-Aldrich. Bovine serum albumin (BSA) and 5, 5′-dimethyl-1-pyrroline-oxide (DMPO) was obtained from Energy Chemical (Shanghai, China). Human serum were purchased from LABLEAD. All solutions were prepared with ultrapure water obtained from a Barnstead Nanopure Water System.

1.2 Characterization of samples

The morphologies of the samples were viewed by field emission scanning electron microscopy (FE-SEM, HITACHI-S4800, Japan), and high-angle annular dark-field scanning TEM (HAADF-STEM) and energy-dispersive X-ray spectroscopy (EDS) elemental mapping images were obtained by transmission electron microscopy (TEM, Tecnai F30, USA, 300 kV). The crystallinity was investigated by powder X-ray diffraction (XRD, Ultima IV) using Cu Kα radiation at λ = 1.5418 Å, 40 kV, and 40 mA. X-ray photoelectron spectroscopy (XPS) was performed on a PHI Quantum-2000 spectrometer. Thermal gravimetric analysis (TGA) was conducted on TA instrument (Q600 SDT, USA) from room temperature to 800 °C under N_2 atmosphere. The hydroxyl radical signals were collected by electron spin resonance (ESR) spectrometry (Bruker X-band A200, German). The absorption spectra of steady-state kinetic measurements were collected on a Shimadzu UV-2550 spectrophotometer (Shimadzu, Japan). Tecan Safire2 Multi-Mode Microplate Reader was employed for other colorimetric measurements. In-situ Fourier transform infrared (FTIR) reflection spectroscopy tests were operated on a Nicolet-8700 spectrometer. The X-ray absorption find structure spectra (Fe K-edge) were

collected at beamline BL44B2 at the SPring-8 synchrotron in Japan. The storage rings of SPring-8 was operated at 8.0 GeV with a maximum current of 250 mA. Using Si (111) double-crystal monochromator, the data collection was carried out in transmission mode using ionization chamber. All spectra were collected in ambient conditions.

1.3 Synthesis of Comparison Sample

Synthesis of Aux/N–C-300: To form L-Cys-Au(I) complexes, aqueous solutions of $HAuCl_4$ (23.4 mM, 0.4 mL) and L-Cys (5 mM, 4 mL) were mixed in water (4.7 mL). An aqueous NaOH solution (1 M, 0.1 mL) was then introduced into the reaction mixture, followed by the addition of 0.2 mL of $NaBH_4$ solution (prepared by dissolving 43 mg of $NaBH_4$ powder in 10 mL of 0.2 M NaOH solution). The L-Cys -Au_{25} NCs were obtained after 3 hours for further synthesis. Subsequently, 25 mg N–C were dissolved in water (5 mL), which was added to the above L-Cys-Au_{25} NCs aqueous solution. After stirring for 20 hours at room temperature, the Au_{25}/N–C was obtained by centrifugation, washed thoroughly with water, and dried at 60 °C under vacuum. The resulting Au_{25}/N–C power was then transferred into a ceramic boat and heated to 300 °C for 2 hours with a heating rate of 5 °C min^{-1} under N_2, followed by natural cooling to room temperature.

Synthesis of L-Cys/Fe–N–C-300: L-Cys (5 mM, 4 mL) were mixed in water (5.4 mL). Subsequently, 25 mg Fe–N–C were dissolved in water (5 mL), which was added to the above L-Cys aqueous solution. After stirring for 20 hours at room temperature, the L-Cys/Fe–N–C was obtained by centrifugation, and dried at 60 °C under vacuum. The resulting L-Cys/Fe–N–C power was then transferred into a ceramic boat and heated to 300 °C for 2 hours with a heating rate of 5 °C min^{-1} under N_2, followed by natural cooling to room temperature.

Synthesis of Au NP/Fe–N–C: aqueous solutions of $HAuCl_4$ (23.4 mM, 0.4 mL) were mixed in water (9 mL). Subsequently, 25 mg Fe–N–C were dissolved in water (5 mL), which was added to the above $HAuCl_4$ aqueous solution. After stirring for 20 hours at room temperature, the Au/Fe–N–C was obtained by centrifugation, and dried at 60 °C under vacuum. The resulting Au/Fe–N–C power was then transferred into a ceramic boat and heated to 300 °C for 2 hours with a heating rate of 5 °C min^{-1} under N_2, followed by natural cooling to room temperature.

Synthesis of Au_{25}: To form L-Cys-Au(I) complexes, aqueous solutions of $HAuCl_4$ (23.4 mM, 0.4 mL) and L-Cys (5 mM, 4 mL) were mixed in water (4.7 mL). An aqueous NaOH solution (1 M, 0.1 mL) was then introduced into the reaction mixture, followed by the addition of 0.2 mL of $NaBH_4$ solution (prepared by dissolving 43 mg of $NaBH_4$ powder in 10 mL of 0.2 M NaOH solution). The L-Cys -Au_{25} NCs were obtained after 3 hours for further synthesis. Excess ligands and ions were removed by dialysis in aqueous solution for 12 hours and then freeze-dried.

1.4 Testing of enzyme-mimetic properties of samples

The enzyme-mimetic catalytic activities of samples were evaluated using TMB substrates on 96-well plates for four times. To confirm the oxidase-like activity, TMB (50 µL, 10 mM) and nanozymes (10 µL, 0.1 mg mL^{-1}) were sequentially added into acetate buffer solution (200 µL, pH=4), and the absorbance were then

recorded at 652 nm by a multi-mode microplate reader after 5 min reaction. Meanwhile, the peroxidase activities were tested at 0.5 mM hydrogen peroxide, which is the same as saturated oxygen concentrations. The production of O_2^- intermediates during the oxidase-like reaction was monitored using ESR under the experimental conditions, with DMPO serving as a spin trap.

Steady-state kinetic measurements: The steady-state kinetic measurements of oxidase-like nanozymes based on $Au_x/Fe\text{-}S_1N_4\text{-}C$ were conducted by recording the absorbance at 652 nm. To this end, different concentrations of TMB were first added into acetate buffer (935 µL) followed by $Au_x/Fe\text{-}S_1N_4\text{-}C$ (30 µL, 0.1 mg mL^{-1}). The absorbance A of each solution was recorded immediately. The resulting substrate concentration-dependent reaction rate curves were then fitted according to the Michaelis-Menten model. The maximum reaction rate (v_{max}) and Michaelis-Menten constant (Km) were calculated using Lineweaver-Burk plot (double-reciprocal plot) shown in Eq. (1):

$$1/v = Km/v_{max} \times 1/[S] + 1/v_{max} \quad (1)$$

where v is the initial velocity and [S] represents the concentration of TMB.

Detection of L-Cys: Based on the inhibition mechanism of nanozymes by sulfhydryl molecules, the $Au_x/Fe\text{-}S_1N_4\text{-}C$ nanozymes + TMB system was used for the detection of L-Cys. Various concentrations of L-Cys solution were added into acetate buffer (pH=4). Subsequently, 10 µL of 0.1 mg mL^{-1} $Au_x/Fe\text{-}S_1N_4\text{-}C$ nanozymes were added and the resulting solution was incubated for 1 min. Next, 50 µL of 10 mM TMB was added, and the solution was incubated at 37 °C for 5 min. The absorbance values at 652 nm were measured for both uninhibited $Au_x/Fe\text{-}S_1N_4\text{-}C$ nanozymes (A_0) and inhibited $Au_x/Fe\text{-}S_1N_4\text{-}C$ nanozymes (A). The relationship between the concentration of L-Cys and $(A_0\text{-}A)/A_0 \times 100$ was obtained for the detection of L-Cys.

Detection of AChE: Based on the above inhibition mechanism, the $Au_x/Fe\text{-}S_1N_4\text{-}C$ nanozymes + TMB system can be also used to evaluate the activity of AChE. Mix different concentrations of AChE with 30 µL of 5mM ATCh. After incubating for 5 min at 37 °C, 5 µL of 0.1 mg mL^{-1} $Au_x/Fe\text{-}S_1N_4\text{-}C$ nanozymes were added and incubated for 1 min to completely inhibit $Au_x/Fe\text{-}S_1N_4\text{-}C$. Subsequently, acetate buffer (pH 4.0) was added, and 50 µL of 10 mM TMB was injected before another incubation for 5 min at 37 °C. As stated above, the absorbance values were recorded as A for further use. Another group without the addition of ATCh was carried out to obtain A_0. A linear relationship between the activity of AChE and $(A_0\text{-}A)/A_0 \times 100$ was established to evaluate AChE activity.

Interference study: To measure the anti-interference ability of the $Au_x/Fe\text{-}S_1N_4\text{-}C$ nanozymes + TMB system, a series of potential interfering substrates were chosen for testing. 5-fold concentrations of potential

interfering substrates, including natural enzymes (CAT, lysozyme, GOx, trypsin, HRP, lipase) metal irons (Na^+, K^+, Mg^{2+}, Zn^{2+}, Ca^{2+}, Ni^{2+}), sugars (glucose, sucrose), amino acid (L-proline, L-isoleucine, glycine), and BSA (bovine serum albumin) were added into the $Au_x/Fe\text{-}S_1N_4\text{-}C$ + TMB system. The absorbance values at 652 nm were recorded to compare the interference effect.

1.5 Computational Detail

Spin-polarization density functional theory (DFT) calculations were performed with the Vienna Ab initio Simulation Package (VASP 6.3.1).[1] The exchange and correlation (XC) part in the Kohn−Sham equation was described by Perdew Burke Ernzerhof (PBE) functional in terms of the gradient of electronic density.[2] The projector-augmented wave (PAW) pseudopotential, featuring the greater computational efficiency of plane wave as well as high accuracy of ultrasoft pseudopotential, was employed in the treatment of the ion-electron interaction. Plane wave function with kinetic energy less than E_{cut} of 400 eV is included in the basic set. The sampling of Brillouin zone adopted the k-mesh of 4×4×1 grid for geometrical optimization calculations and electronic properties calculations. For self-consistent field (SCF) calculations, the stopping criterion was set to the energy difference less than 1.0×10^{-5} eV, while for geometrical optimization the convergence will reach until the Hellmann−Feynman force per atom less than 0.02 eV/Å. Moreover, DFT-D3(BJ) method was used to correct the Van der Waals force.[3] To correct the field Coulomb interaction in DFT calculation, U = 4.48 eV for Fe 3d state by the following formula:[4]

$$U \approx \left(\frac{\partial N_{SCF}}{\partial V}\right)^{-1} - \left(\frac{\partial N_{NSCF}}{\partial V}\right)^{-1} \qquad (1)$$

Moreover, the adsorption energy was typically estimated by following:

$$E_{ads} = E(\text{reactant/ catalyst}) - E(\text{reactant}) - E(\text{catalyst}) \qquad (2)$$

where E(reactant/catalyst), E(reactant) and E(catalyst) represents the energy of relaxed configuration of catalyst, reactant and reactant-adsorbed catalyst.

In addition, for each elemental step, the Gibbs free energies (G) was calculated by following equation (3):

$$\Delta G = \Delta E_{DFT} + \Delta ZPE + \Delta H - T\Delta S \qquad (3)$$

where E_{DFT} was the total energy calculated by VASP, ΔZPE and TΔS were zero-point energy and entropy contributions calculated from VASPKIT.[5] T stands for temperature and was set to 298.15 K and H was the heat capacity.

2. Supplementary Results

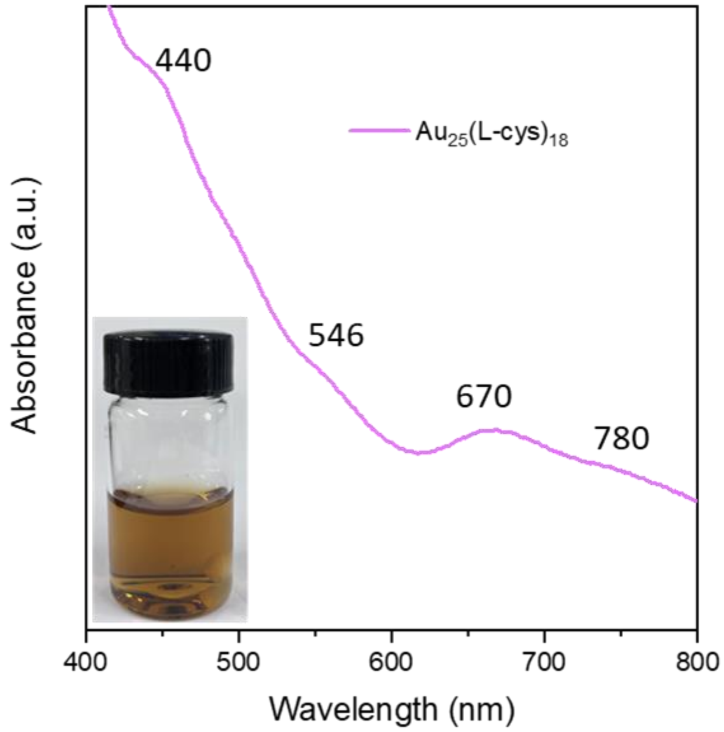

Figure S1. UV-Vis absorption spectrum of $Au_{25}(L\text{-}Cys)_{18}$ clusters.

The fingerprint peaks at 780, 670, 546, and 440 nm of Au_{25} clusters were detected in UV-vis absorption spectrum, which is consistent with the result of reported $Au_{25}(L\text{-}Cys)_{18}$ nanoclusters.[6] This indicates confirmed that $Au_{25}(L\text{-}Cys)_{18}$ nanoclusters was successfully synthesized.

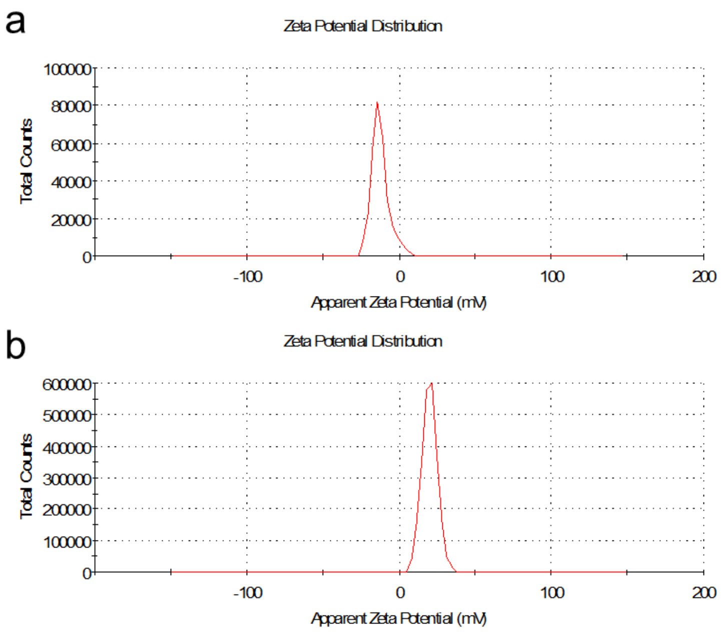

Figure S2. Zeta potential of (a) $Au_{25}(L\text{-}Cys)_{18}$ clusters and (b) Fe@ZIF-8 derived Fe-N-C matrix.

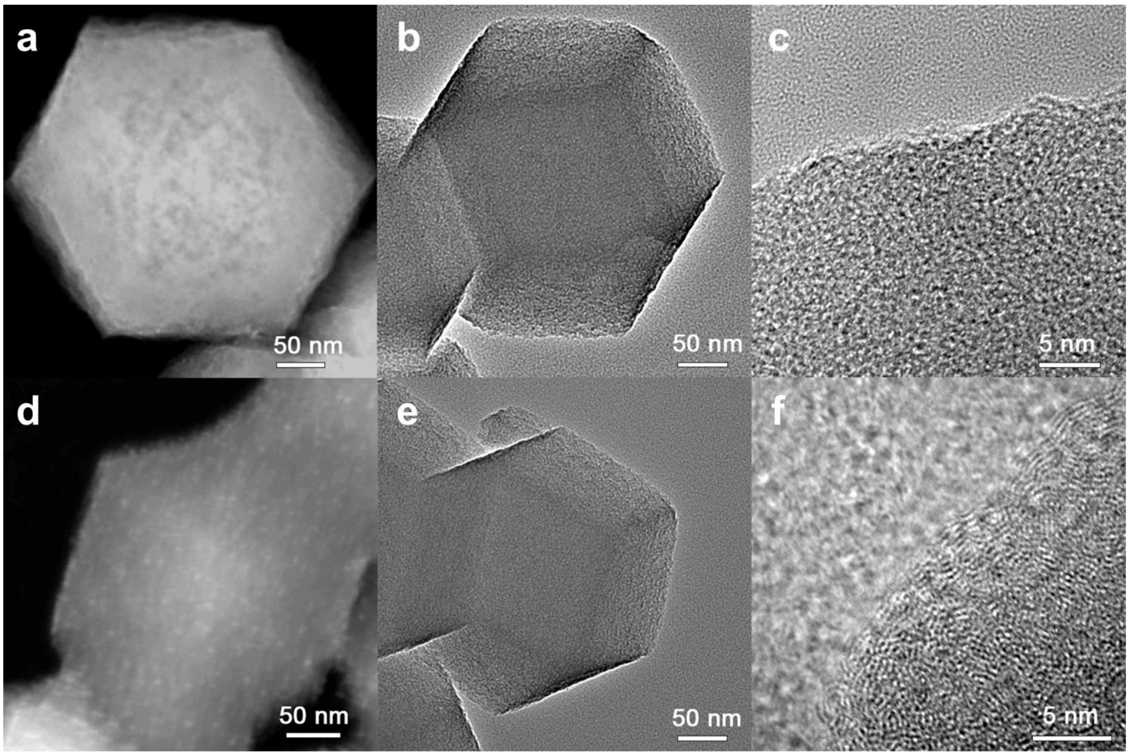

Figure S3. HADDF-STEM and TEM images of (a-c) Fe-N-C and (d-f) Au_{25}/Fe-N-C without undergoing re-pyrolysis.

Compared to Fe-N-C (Figure S3a), Au_{25}/Fe-N-C (Figure S3d) has some small metal clusters indicating successful loading of Au_{25}. However, the observation is not satisfactory due to the small size of the gold clusters (~1.5 nm), lack of crystallinity and the presence of ligands.

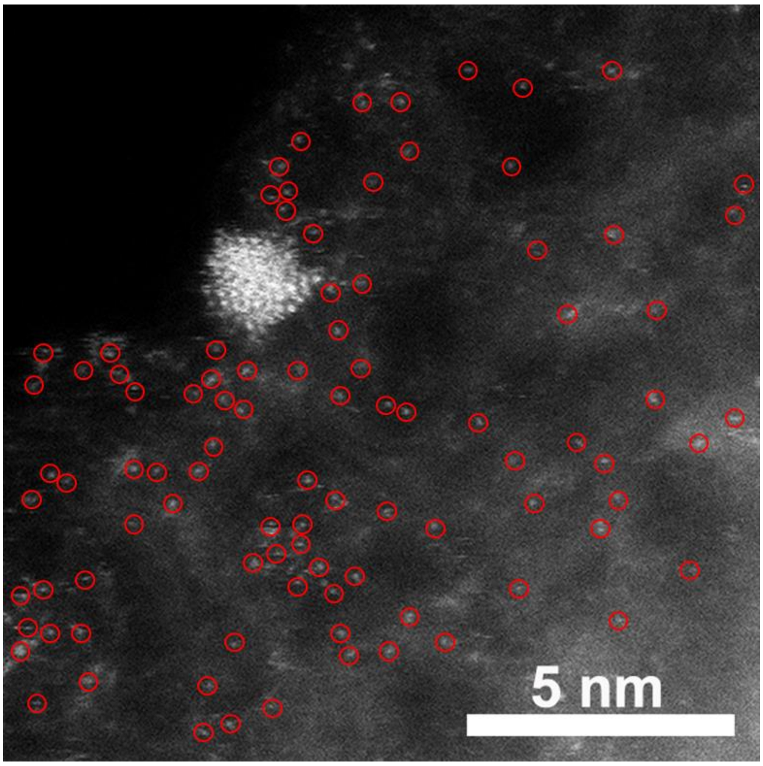

Figure S4. AC-HAADF-STEM image of Au_x/Fe-S_1N_4-C.

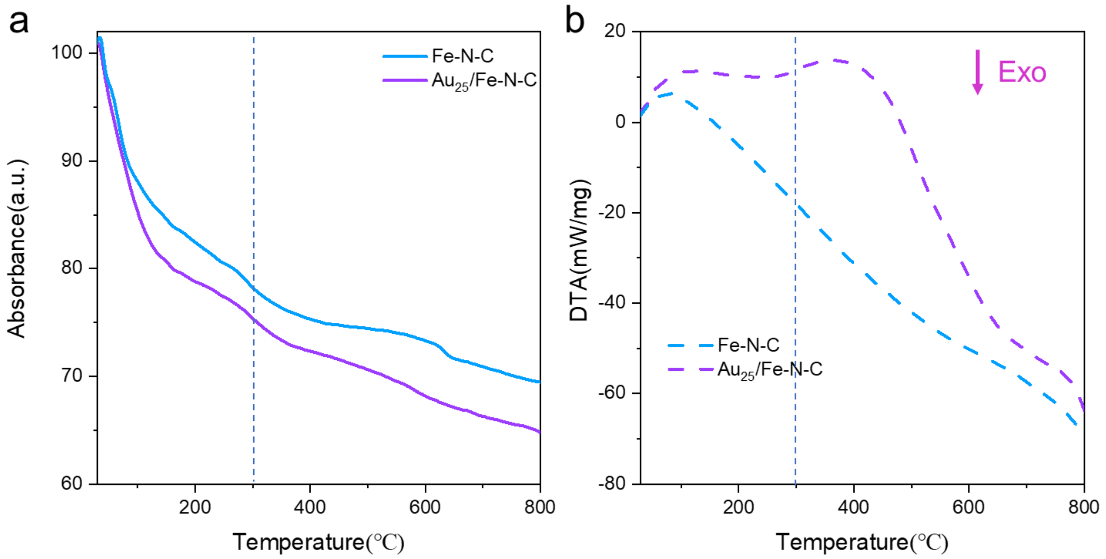

Figure S5. (a) TGA and (b) DTA curves of Fe-N-C and Au_{25}/Fe-N-C under N_2 flow.

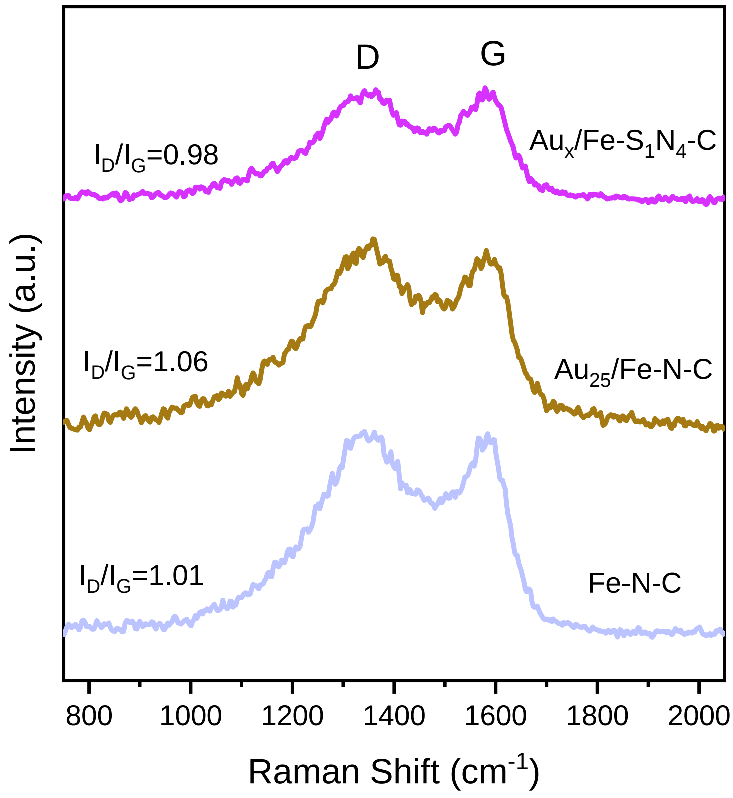

Figure S6. Raman spectra of Au_x/Fe-S_1N_4-C, Au_{25}/Fe-N-C, and Fe-N-C.

Compared to the pristine Fe-N-C, the I_D/I_G for the Au_x/Fe-S_1N_4-C was slightly reduced, indicating the degree of graphitization of Fe-N-C matrix was improved after secondary pyrolysis to some extent.

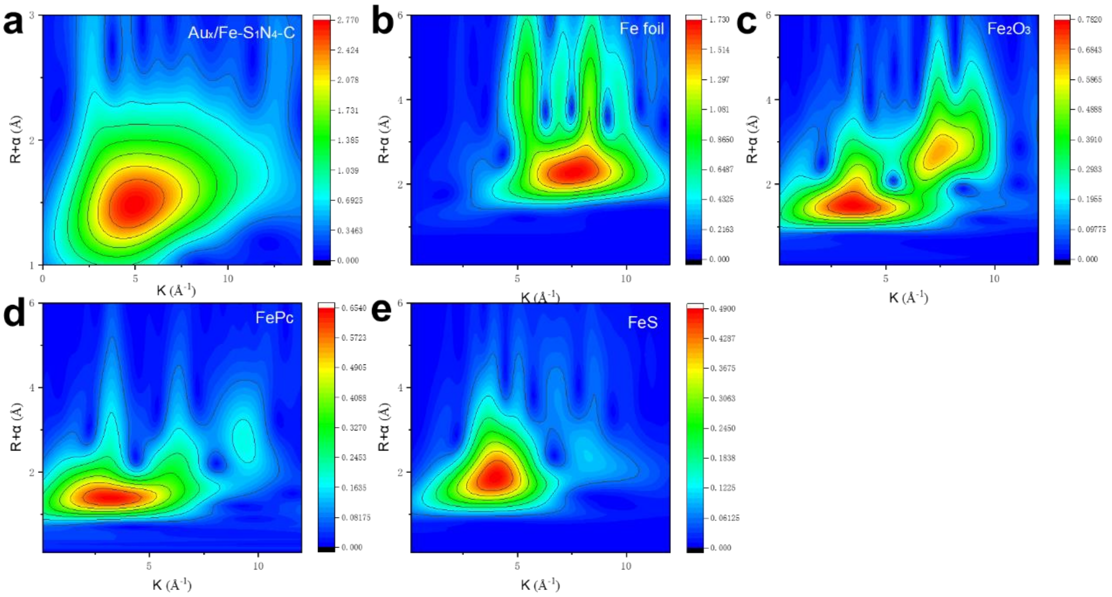

Figure S7. Wavelet transforms signals at Fe K-edge for (a) Au_x/Fe-S_1N_4-C, (b) Fe foil, (c) Fe_2O_3, (d) FePc, and (e) FeS.

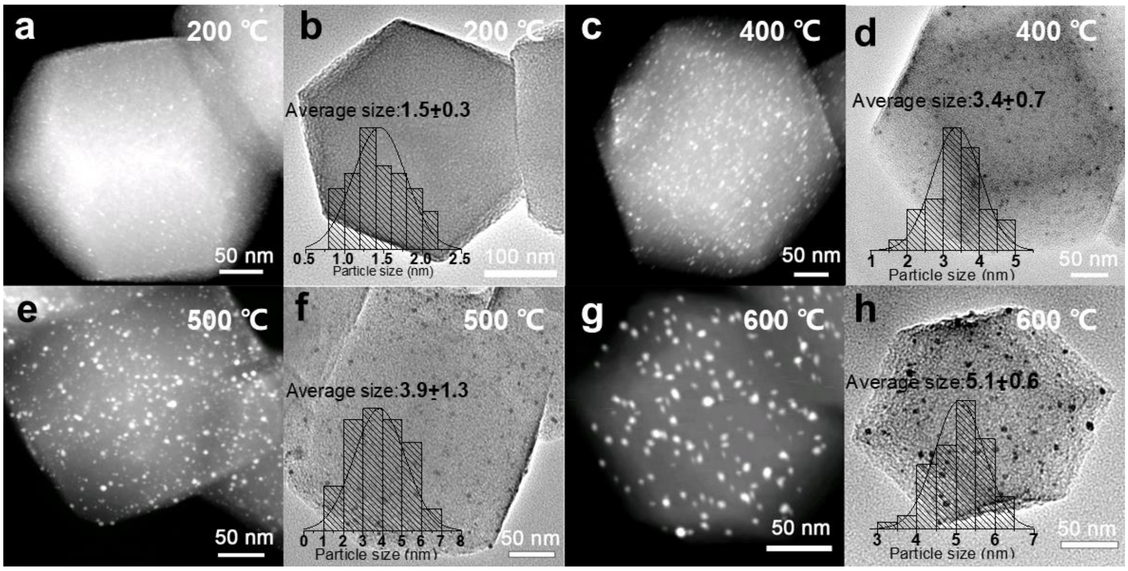

Figure S8. HADDF-STEM and TEM images of Au_x/Fe-N-C-200, Au_x/Fe-N-C-400, Au_x/Fe-N-C-500 and Au_x/Fe-N-C-600. The insets are the corresponding particle-size histograms.

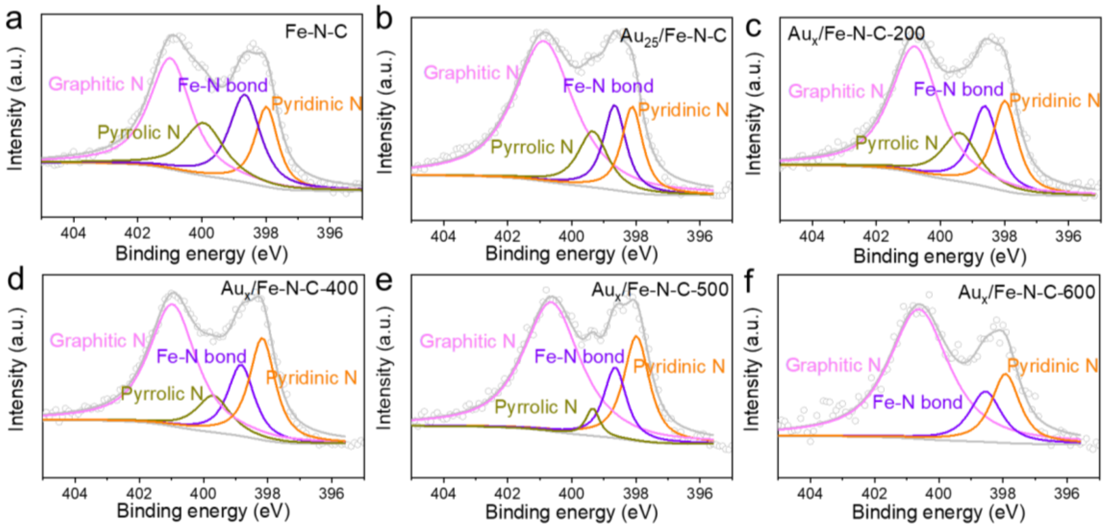

Figure S9. High-resolution XPS spectra of N 1s of Fe-N-C, Au_{25}/Fe-N-C, Au_x/Fe-N-C-200, Au_x/Fe-N-C-400, Au_x/Fe-N-C-500 and Au_x/Fe-N-C-600.

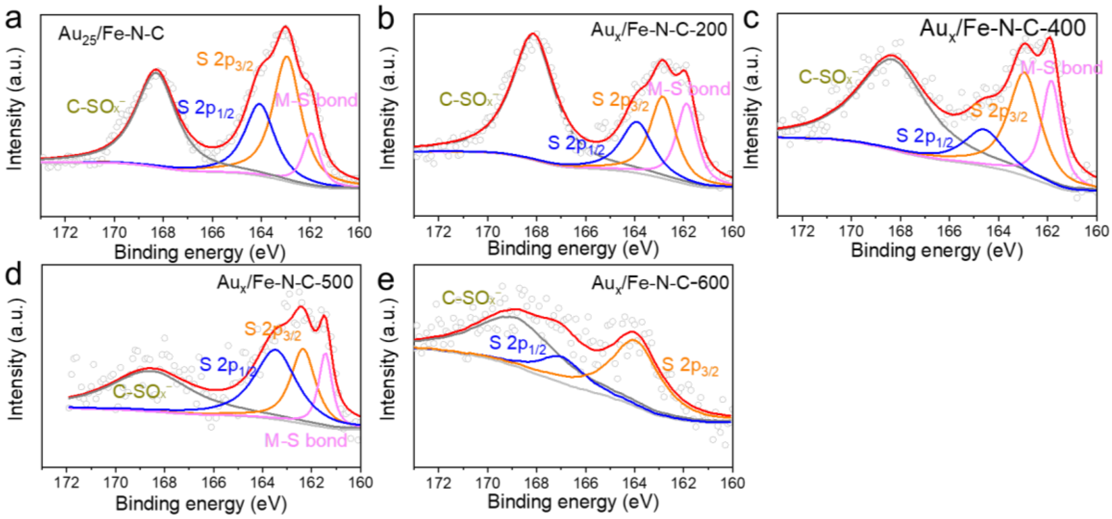

Figure S10. High-resolution XPS spectra of S 2p of Au_{25}/Fe-N-C, Au_x/Fe-N-C-200, Au_x/Fe-N-C-400, Au_x/Fe-N-C-500 and Au_x/Fe-N-C-600.

Of note, high temperature would cause the loss of ligands. There were no metal-S bonds detected in the Au_x/Fe-N-C-600, indicating that the sulfhydryl ligands were completely lost at 600 ℃.

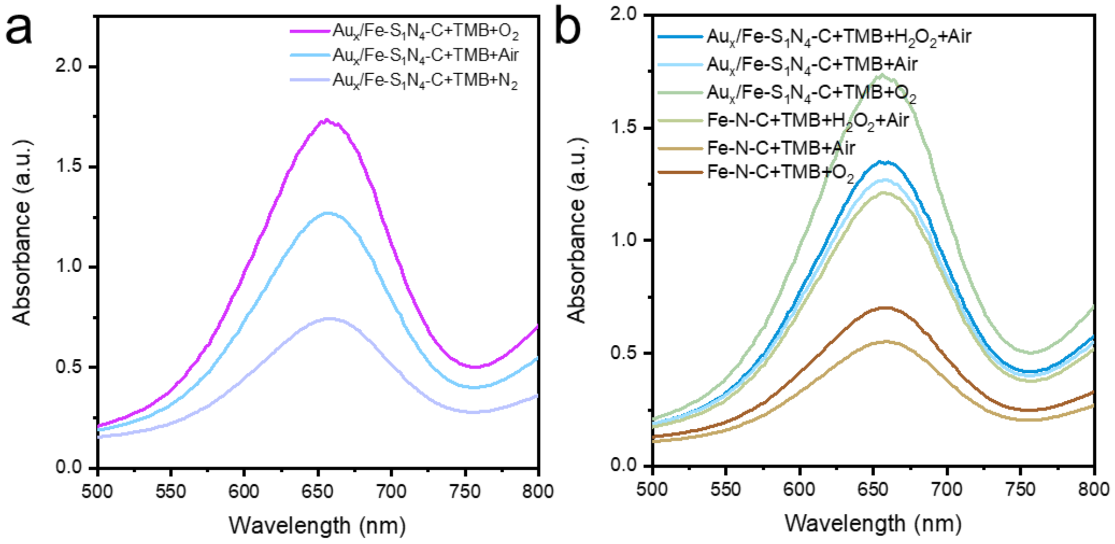

Figure S11. (a) UV-vis spectra of $Au_x/Fe-S_1N_4-C$ in the conditions of O_2-saturated, air and N_2-saturated. (b) UV-vis of peroxidase-like and oxidase-like activities of $Au_x/Fe-S_1N_4-C$ and Fe-N-C. Note: In order to eliminate the interference of dissolved oxygen in the peroxidase test, the absorbance of Fe-N-C and $Au_x/Fe-S_1N_4-C$ samples in the air was deducted from the absorbance added with hydrogen peroxide to obtain the absorbance change caused by pure hydrogen peroxide (Figure 3a).

To examine the effect of oxygen concentration, the oxidase-like nanozyme property of $Au_x/Fe-S_1N_4-C$ was measured in O_2, air, and N_2 saturated NaAc buffer medium solutions with the optimal pH of 4 (Figure S10). It can be seen that the oxidation rate of 3,3',5,5'-tetramethylbenzidine (TMB) is highly correlated with O_2 concentration, which reveals that $Au_x/Fe-S_1N_4-C$ can catalyze the decomposition of O_2 into reactive oxygen species.

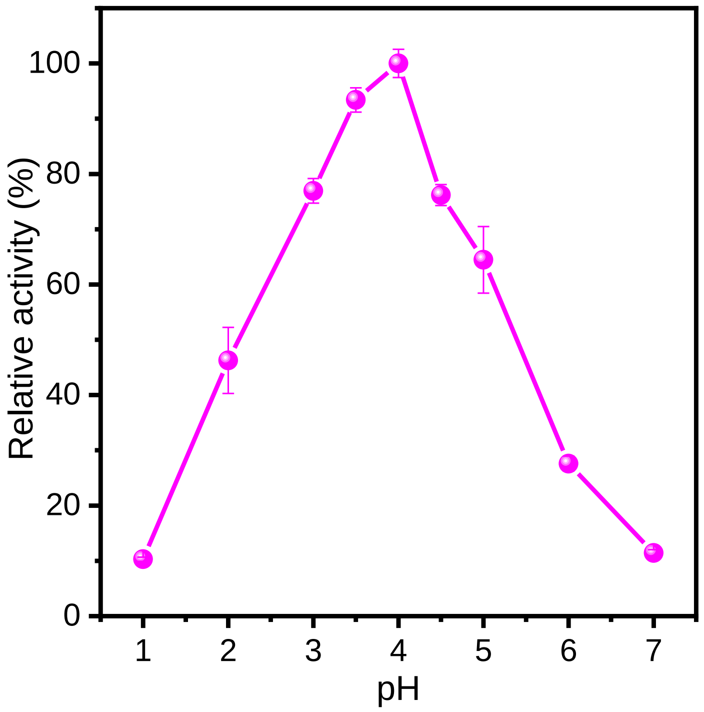

Figure S12. Influence of pH on the oxidase-like activity of Au_x/Fe-S_1N_4-C.

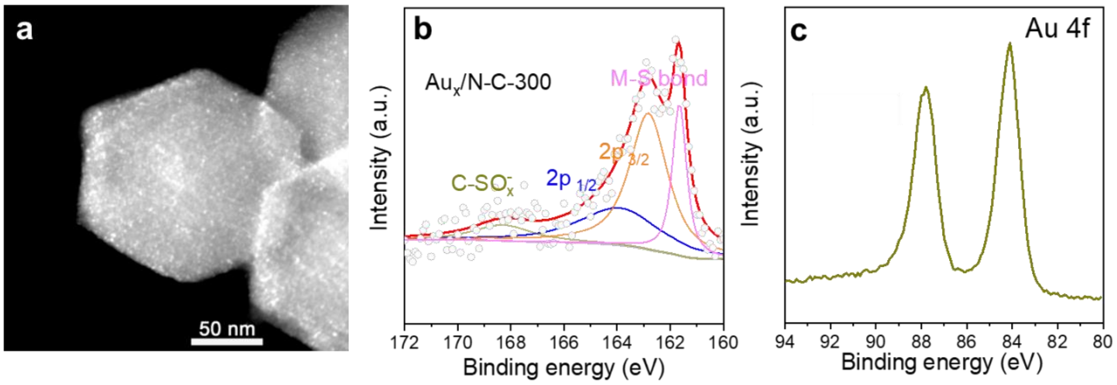

Figure S13. Au_x/N-C-300: (a) HADDF-STEM image, (b) High-resolution XPS spectra of S 2p, and (c) High-resolution XPS spectra of Au 4f.

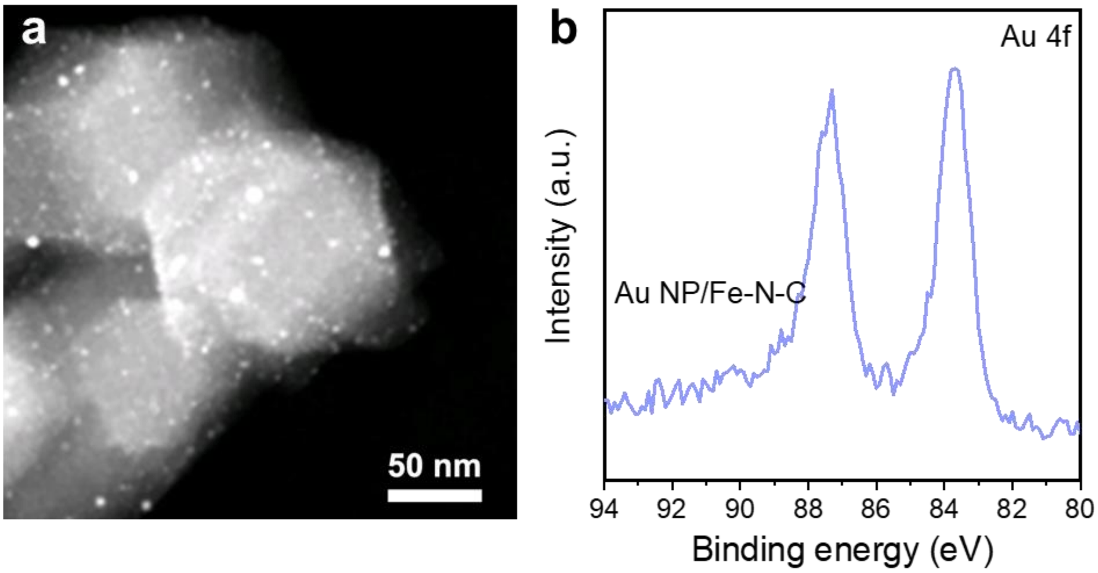

Figure S14. Au NP/Fe-N-C: (a) HADDF-STEM image, and (b) High-resolution XPS spectra of Au 4f.

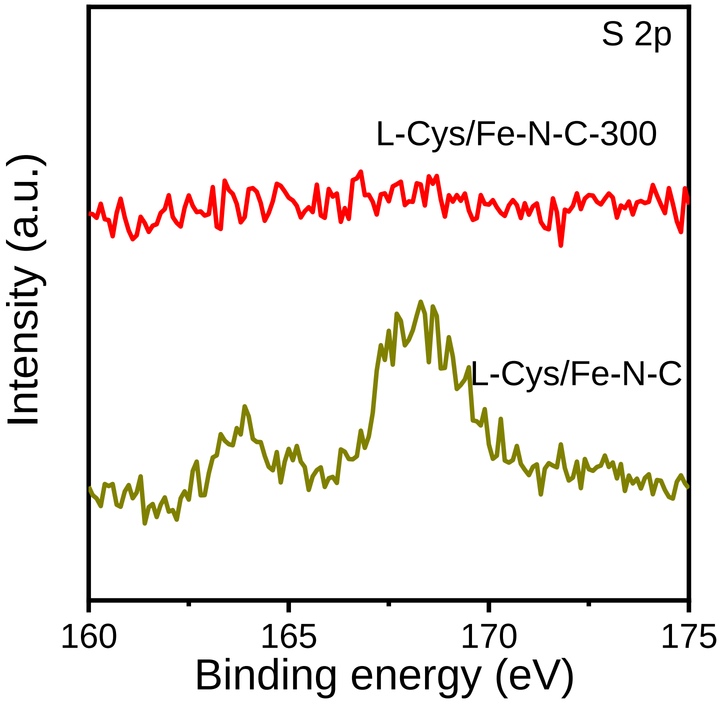

Figure S15. High-resolution XPS spectra of S 2p of L-Cys/Fe-N-C and L-Cys/Fe-N-C-300.

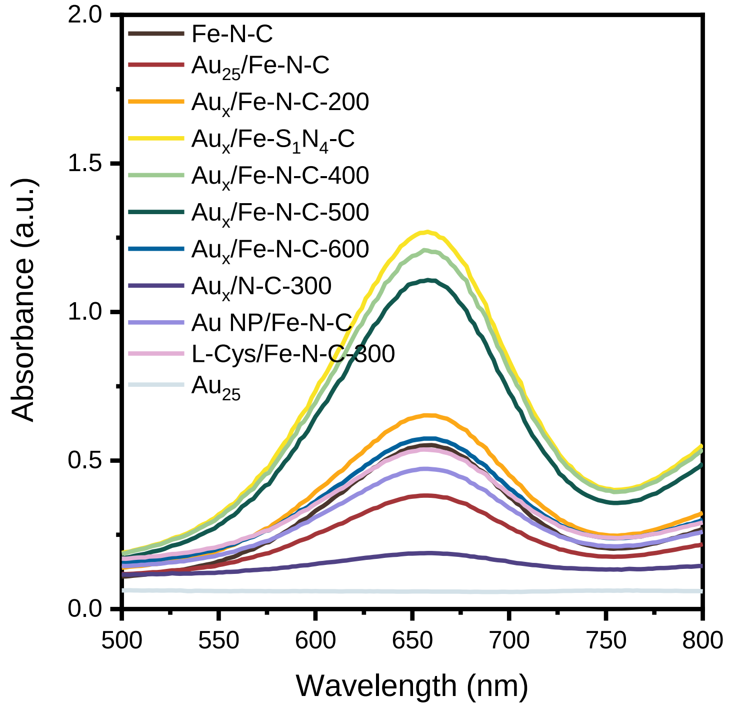

Figure S16. UV-vis spectra of TMB solutions containing different samples.

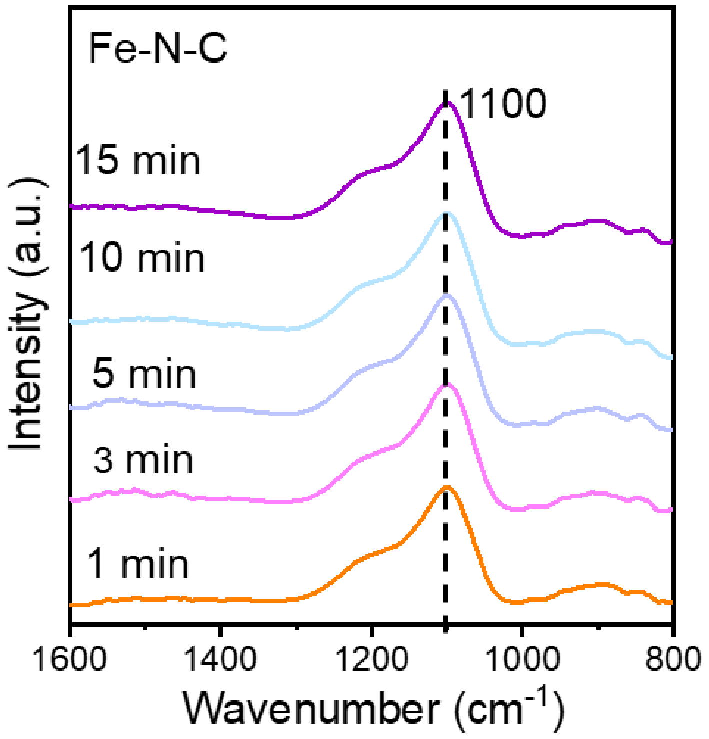

Figure S17. In situ FTIR recorded under an O_2 atmosphere with Fe-N-C.

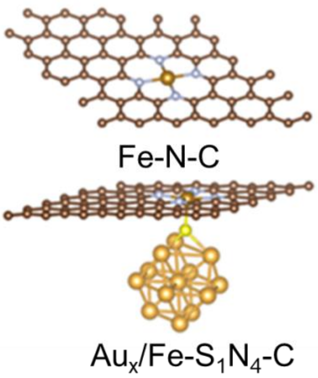

Figure S18. The atomic structures of Fe-N-C and Au_x/Fe-S_1N_4-C were optimized.

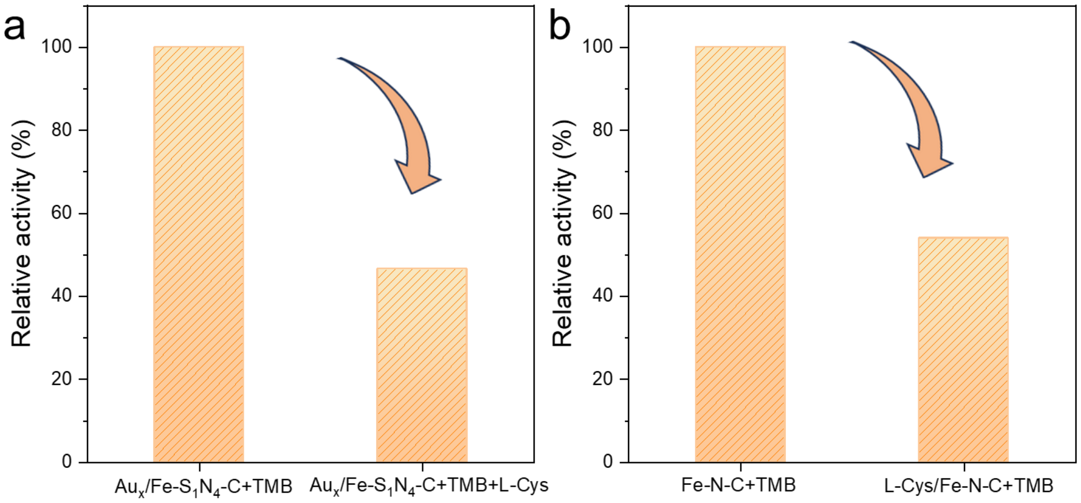

Figure S19. The relative activity of different samples.

To investigate the role of L-Cys, we conducted two comparative experiments. First, TMB and Au_x/Fe-S_1N_4-C reacted for 5 minutes, and the absorbance of the resulting oxTMB was recorded. Then, L-Cys was added and incubated for 1 minute, followed by measuring the absorbance again. By comparing the changes in absorbance, we could determine whether L-Cys functions as a reducing agent.

In the second experiment, we demonstrated the toxic effect of L-Cys. To assess the toxicity, Fe-N-C was used as a representative sample instead of Au_x/Fe-S_1N_4-C, as the S content in the latter was not suitable for observing the physical loading of L-Cys. First, the active sites of Fe-N-C are occupied by physically adsorbing L-Cys (the successful loading of L-Cys was confirmed by XPS, as shown in Figure S16). After 5 minutes of reaction with TMB, the absorbance was measured. We observed a reduced absorption compared to that measured by Fe-N-C.

These two experiments provide evidence that L-Cys exhibits both reducing properties and the ability to poison the active site of the nanozymes.

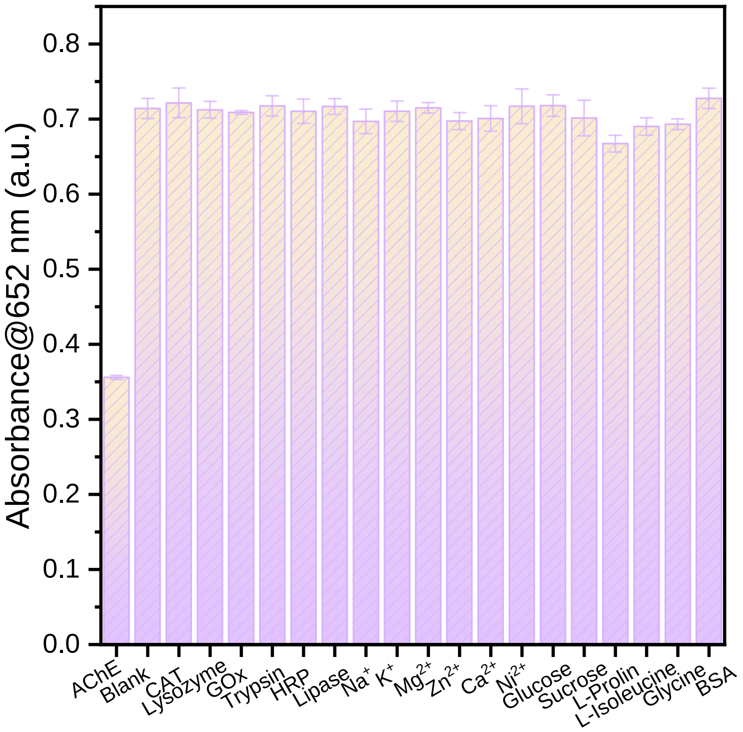

Figure S20. The selectivity of AChE detection with different interference.

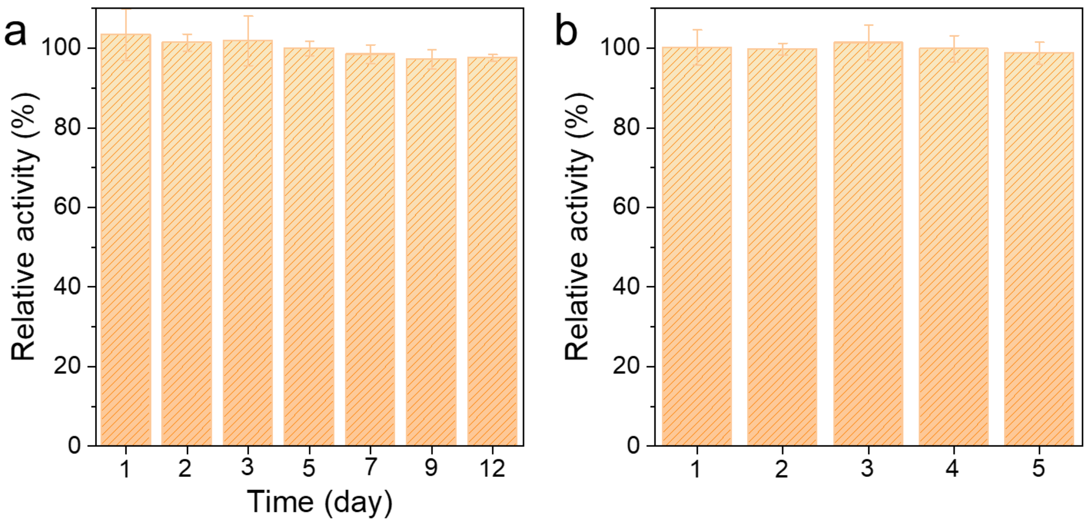

Figure S21. (a) Stability of AChE detection by Au_x/Fe-S_1N_4-C nanozymes. (b) Reproducibility of five Au_x/Fe-S_1N_4-C nanozyme-based AChE biosensors for detecting 5 mU/mL AChE.

Table S1. EXAFS fitting parameters at the Fe K-edge for Au_x/Fe-S_1N_4-C.

Sample	Path	N	R (Å)	σ^2 ($\times 10^{-3}$ Å^2)	E_0 (eV)	R-factor
Au_x/Fe-S_1N_4-C	Fe-N	3.88	1.83	5.86	3.71	0.006
	Fe-S	0.66	2.14	2.40	3.71	0.006

N: coordination numbers; *R*: bond distance; σ^2: Debye-Waller factors; ΔE_0: the inner potential correction. *R* factor: goodness of fit.

Table S2. Atomic percentage of all the samples determined by XPS.

Nanozyme	C (at%)	N (at%)	S (at%)	Fe (at%)	Au (at%)
Fe-N-C	96.23	3.47	0	0.29	0
Au_{25}/Fe-N-C	92.41	4.52	2.70	0.04	0.33
Au_x/Fe-N-C-200	93.72	4.19	1.60	0.23	0.25
Au_x/Fe-S_1N_4-C	93.02	4.05	2.39	0.29	0.26
Au_x/Fe-N-C-400	93.33	5.16	0.89	0.40	0.33
Au_x/Fe-N-C-500	94.76	3.89	0.92	0.28	0.16
Au_x/Fe-N-C-600	96.36	2.24	1.00	0.35	0.04

Table S3. Fitting results of N1s for all the nanozymes assessed by XPS.

Nanozyme	graphitic-N	pyrrolic-N	metal-N	pyridinic-N
Fe-N-C	41.0	18.7	24.1	16.2
Au_{25}/Fe-N-C	61.0	12.1	13.7	13.2
Au_x/Fe-N-C-200	49.9	14.5	17.4	18.2
Au_x/Fe-S_1N_4-C	52.7	13.0	19.2	15.1
Au_x/Fe-N-C-400	50.0	10.7	16.4	22.9
Au_x/Fe-N-C-500	61.1	3.2	13.3	22.4
Au_x/Fe-N-C-600	68.5	0.0	14.2	17.3

Table S4. Fitting results of S 2p for all the nanozymes determined by XPS.

Nanozyme	$C\text{-}SO_X^-$	S 2p	metal-S
Au_{25}/Fe-N-C	36.8	54.5	8.7
Au_x/Fe-N-C-200	49.5	34.9	15.6
Au_x/Fe-S_1N_4-C	11.6	66.0	22.4
Au_x/Fe-N-C-400	48.0	36.4	15.6
Au_x/Fe-N-C-500	23.2	61.6	11.1
Au_x/Fe-N-C-600	47.3	52.7	0.0

Table S5. Comparison of nanozyme-based biosensors for the detection of AChE.

Nanozyme	Method	Linear range ($mU\ mL^{-1}$)	LOD ($mU\ mL^{-1}$)	Reference
$Au_x/Fe\text{-}S_1N_4\text{-}C$	Colormetric	1 – 10	0.0051	This work
Ag^+@CTAB-AuNPs	Colormetric	0.075 – 25	0.075	*Small* **2018**, *14*, e1801680.[7]
Au@PDA NPs hydrogel	Colormetric	2.5 – 25	0.9	*Anal. Chem.* **2018**, *90*, 11423–11430.[8]
PAA-CeO_2	Fluorescence	0.263 – 50	0.263	*Biosens. Bioelectron.* **2016**, *85*, 457-463.[9]
Citrate-CeO_2	Colormetric	0 – 1400	3.5	*ACS Sens.* **2016**, *1*, 1336–1343.[10]
AChE-MnO_2-TMB	Colormetric	0.1 – 15	0.035	*Nanoscale* **2017**, *9*, 2317–2323.[11]
P,O-g-C_3N_4 nanodots	Fluorescence	0.01 – 3	0.01	*J. Mater. Chem. C* **2015**, *3*, 10916-10924.[12]
PhO-dex-GO	Fluorescence	0.1 – 100	0.27	*ACS Nano* **2016**,*10*, 5346-5353.[13]
Fe-N-C SAzymes	Colormetric	0.5 – 25	0.014	*Small* **2019**, *15*, e1903108.[14]

Table S6. Detection of L-Cys in diluted human serum based on the standard addition method.

Sample	Spiked (μM)	Measured (μM)	Recovery (%)	RSD (%, n=3)
1	16.67	16.61	99.66	0.59
	33.33	33.37	100.11	0.15
	50.00	50.11	100.21	0.22
	66.67	66.65	99.98	0.17
2	16.67	16.63	99.78	0.13
	33.33	33.31	99.95	0.60
	50.00	50.08	100.15	0.34
	66.67	66.69	100.03	0.26

Table S7. Results of AChE activity assay in diluted human serum based on the standard addition method.

Sample	Spiked (mU mL^{-1})	Measured (mU mL^{-1})	Recovery (%)	RSD (%, n=3)
1	1.00	1.01	101.00	1.98
	2.50	2.50	100.05	0.82
	5.00	5.04	100.82	1.28
2	1.00	1.01	101.10	1.24
	2.50	2.49	99.67	1.17
	5.00	5.00	100.09	0.25

References

[1] a) G. Kresse, J. Hafner, *Phys. Rev. B* **1993**, *47*, 558-561; b) G. Kresse, J. Hafner, *Phys. Rev. B* **1994**, *49*, 14251-14269; c) G. Kresse, J. Furthmiiller, *Comput. Mater. Sci.* **1996**, *6*, 15-50; d) G. Kresse, J. Furthmuller, *Phys. Rev. B* **1996**, *54*, 11169-11186.

[2] J. P. Perdew, K. Burke, M. Ernzerhof, *Phys. Rev. Lett.* **1996**, *77*, 3865-3868.

[3] S. Grimme, J. Antony, S. Ehrlich, H. Krieg, *J. Chem. Phys.* **2010**, *132*, 154104.

[4] M. Cococcioni, S. de Gironcoli, *Phys. Rev. B* **2005**, *71*, 035105.

[5] V. Wang, N. Xu, J.-C. Liu, G. Tang, W.-T. Geng, *Comput. Phys. Commun.* **2021**, *267*, 108033.

[6] X. Yuan, B. Zhang, Z. Luo, Q. Yao, D. T. Leong, N. Yan, J. Xie, *Angew. Chem. Int. Ed.* **2014**, *53*, 4623-4627; *Angew. Chem.* **2014**, *126*, 4711-4715.

[7] J. Zhang, W. Zheng, X. Jiang, *Small* **2018**, *14*, e1801680.

[8] J. Zhang, L. Mou, X. Jiang, *Anal. Chem*. **2018**, *90*, 11423-11430.

[9] S. X. Zhang, S. F. Xue, J. Deng, M. Zhang, G. Shi, T. Zhou, *Biosens. Bioelectron*. **2016**, *85*, 457-463.

[10] H. Cheng, S. Lin, F. Muhammad, Y.-W. Lin, H. Wei, *ACS Sens*. **2016**, *1*, 1336-1343.

[11] X. Yan, Y. Song, X. Wu, C. Zhu, X. Su, D. Du, Y. Lin, *Nanoscale* **2017**, *9*, 2317-2323.

[12] M. Rong, X. Song, T. Zhao, Q. Yao, Y. Wang, X. Chen, *J. Mater. Chem. C* **2015**, *3*, 10916-10924.

[13] T. W. Kang, S. J. Jeon, H. I. Kim, J. H. Park, D. Yim, H. R. Lee, J. M. Ju, M. J. Kim, J. H. Kim, *ACS Nano* **2016**, *10*, 5346-5353.

[14] Y. Wu, L. Jiao, X. Luo, W. Xu, X. Wei, H. Wang, H. Yan, W. Gu, B. Z. Xu, D. Du, Y. Lin, C. Zhu, *Small* **2019**, *15*, e1903108.
